# Brassinin Inhibits Proliferation in Human Liver Cancer Cells via Mitochondrial Dysfunction

**DOI:** 10.3390/cells10020332

**Published:** 2021-02-05

**Authors:** Taeyeon Hong, Jiyeon Ham, Jisoo Song, Gwonhwa Song, Whasun Lim

**Affiliations:** 1 Department of Food and Nutrition, College of Science and Technology, Kookmin University, Seoul 02707, Korea; taeyeon97@kookmin.ac.kr (T.H.); js_song97@kookmin.ac.kr (J.S.); 2 Institute of Animal Molecular Biotechnology, Department of Biotechnology, College of Life Sciences and Biotechnology, Korea University, Seoul 02841, Korea; glorijy76@korea.ac.kr

**Keywords:** brassinin, liver cancer, apoptosis, ROS, mitochondria

## Abstract

Brassinin is a phytochemical derived from Chinese cabbage, a cruciferous vegetable. Brassinin has shown anticancer effects on prostate and colon cancer cells, among others. However, its mechanisms and effects on hepatocellular carcinoma (HCC) have not been elucidated yet. Our results confirmed that brassinin exerted antiproliferative effects by reducing proliferating cell nuclear antigen (PCNA) activity, a proliferation indicator and inducing cell cycle arrest in human HCC (Huh7 and Hep3B) cells. Brassinin also increased mitochondrial Ca^2+^ levels and depolarized the mitochondrial membrane in both Huh7 and Hep3B cells. Moreover, brassinin generated high amounts of reactive oxygen species (ROS) in both cell lines. The ROS scavenger N-acetyl-L-cysteine (NAC) inhibited this brassinin-induced ROS production. Brassinin also regulated the AKT and mitogen-activated protein kinases (MAPK) signaling pathways in Huh7 and Hep3B cells. Furthermore, co-administering brassinin and pharmacological inhibitors for JNK, ERK1/2 and P38 decreased cell proliferation in both HCC cell lines more than the pharmacological inhibitors alone. Collectively, our results demonstrated that brassinin exerts antiproliferative effects via mitochondrial dysfunction and MAPK pathway regulation on HCC cells.

## 1. Introduction

Hepatocellular carcinoma (HCC) is one of the deadliest common cancers worldwide [1]. HCC usually stems from chronic inflammatory conditions and is therefore closely associated with toxic exposures such as chronic viral hepatitis and alcohol [2]. Surgical resection and transplantation are the usual therapeutic methods against HCC. However, these methods do not counter recurrence and the development of resistance to standard chemotherapeutic agents such as sorafenib [3]. Despite the numerous attempts to cure HCC in the past decades, current therapeutics still have limitations and side effects that impair the patients’ quality of life. To overcome these limitations, one strategy is to combine new agents from natural compounds with standard therapy to reduce adverse effects [4]. For instance, celastrol, extracted from vine tree roots, enhanced the tumor suppression and apoptosis induction effects of sorafenib [5].

Phytoalexins are natural compounds produced by various fruits and vegetables in response to infection or stress. Compounds from this family exhibited effects on apoptosis, proliferation, cell cycle progression and migration abilities [6,7,8]. For instance, the well-known resveratrol, curcumin and quercetin revealed anticancer effects on various cancers, including HCC [9]. Brassinin is a phytoalexin found in cruciferous vegetables and has antifungal activities as well as anticancer effects. For instance, brassinin induced DNA fragmentation in human colon cancer and induced mitochondrial apoptosis and reduced anti-apoptotic protein expression in prostate cancer [10,11]. Also, the indole-based structure of brassinin makes it an inhibitor of indoleamine 2,3-dioxygenase (IDO) [12]. This might be helpful against HCC because inhibiting IDO is crucial for blocking cancer immune escape [13,14]. Despite being a potential anticancer agent, the effects and molecular mechanisms of brassinin on HCC remain unexplored.

In this study, we proved that brassinin could regulate cell proliferation and apoptosis in HCC cell lines. We verified the effects of brassinin on both Huh7 and Hep3B cells on (1) cell proliferation and cell cycle progression, (2) mitochondrial dysfunction, (3) generation of reactive oxygen species (ROS) with or without NAC and (4) regulation of phosphatidylinositol 3-kinase (PI3K)/mitogen-activated protein kinases (MAPK) signaling pathway.

## 2. Materials and Methods

### 2.1. Chemicals and Antibodies

Brassinin (catalog no. SML 1635) was purchased from Sigma-Aldrich (St. Louis, MO, USA) and dissolved in dimethyl sulfoxide (DMSO). The antibodies used in the immunoblotting assays are listed in Table 1.

### 2.2. Cell Maintenance of Huh7 and Hep3B

Huh7 and Hep3B cells (HCC cell lines) were purchased from the Korean Cell Line Bank (Seoul, Korea). Huh7 cells were maintained in RPMI1640 with HEPES medium and Hep3B in DMEM/High glucose medium, both containing 10% fetal bovine serum. Monolayer cultures of Huh7 and Hep3B cells were incubated at 37 °C in an incubator with an atmosphere of 5% CO_2_. When cell confluency reached 70% in 60 mm culture dishes or 96-well plates, the cells were incubated in serum-free medium for 24 h and then treated with various concentrations of brassinin for 48 h. AML-12 cells (mouse normal liver cell line) were purchased from American Type Culture Collection (ATCC, Manassas, VA, USA) and cultured according to the manufacturer’s instructions.

### 2.3. Cell Proliferation Assay

The BrdU Cell Proliferation ELISA kit (catalog no. 11647229001, Roche, Basel, Switzerland) was used to assess the proliferation of Huh7 and Hep3B cells. The cells were seeded in 96-well plates and then treated with brassinin (0, 10, 20, 50 and 100 µM) for 48 h in a 37 °C incubator with 5% CO_2_. For the MAPK pathways experiments, the cells were incubated with 20 µM of MAPK pharmacological inhibitor (SP600125, U0126 or SB203580) and with or without 100 µM brassinin for 48 h in a 37 °C incubator with 5% CO_2_. Then, BrdU was added. After incubation with BrdU in a 37 °C incubator with 5% CO_2_ for 2 h, the cells were fixed with a fixation solution and exposed to an anti-BrdU-peroxidase (POD) working solution for 90 min at room temperature. After washing steps, substrate solutions were added to cells and the absorbance (370 and 492 nm) was measured with an ELISA reader. The viability of AML-12 was detected using Cell Proliferation Kit I (MTT) (catalog no. 11465007001, Roche). Experimental procedures were performed according to the manufacturer’s instructions. Fluorescence was detected at 560 nm and 650 nm using an ELISA reader.

### 2.4. Immunofluorescence Microscopy Analysis

The expression of cell nuclear antigen (PCNA) was quantified by immunofluorescence. The cells were seeded in confocal dishes and treated as in Section 2.3. The cells were then fixed with methanol and incubated with mouse monoclonal anti-human PCNA antibody (catalog no. sc-56, Santa Cruz Biotechnology, Santa Cruz, CA, USA). We then incubated the cells with goat anti-mouse IgG Alexa 488 in an antibody dilution buffer. After washing the cells, we stained them with 5 µg/mL DAPI (4′, 6-diamidino-2-phenylindole). We imaged the cells using an LSM710 confocal microscope (Carl Zeiss, Oberkochen, Germany).

### 2.5. Detection of Cell Cycle Arrest

Cells at 60–70% confluence were treated with increasing doses of brassinin for 48 h. The cells were then collected, fixed with 70% ethanol, washed and incubated with RNase A and propidium iodide for 30 min at room temperature. The stained cells were analyzed by flow cytometry.

### 2.6. Detection of Intramitochondrial Intracellular Calcium Level

Huh7 and Hep3B cells were collected after treatment with brassinin and stained with 3 µM of Rhod-2 for 30 min at 4 °C as previously described [15]. The cells were then incubated with Hank’s balanced salt solution (Gibco) for 10 min at 37 °C. The stained cells were analyzed by flow cytometry.

### 2.7. Detection of Depolarization of the Mitochondrial Membrane

A mitochondrial staining kit was used to detect the relative mitochondrial membrane potential (MMP) status in brassinin-treated cells. The cells were treated with increasing doses of brassinin and stained with JC-1 in a staining solution. After staining as previously described [16], the cells were washed with a staining buffer. The stained cells were analyzed by flow cytometry.

### 2.8. Measurement of Cellular ROS

To confirm the generation of ROS, we used 2′,7′-dichlorofluorescein diacetate (DCFH-DA, Sigma-Aldrich). The collected cells were resuspended in a DCFH-DA-containing staining solution. After staining, the cells were washed with phosphate buffer saline and treated with brassinin (0, 10, 20, 50 and 100 µM), NAC (0.5 mM) or both. After several washing steps, the stained cells were analyzed by flow cytometry.

### 2.9. Immunoblotting

Huh7 and Hep3B cells were treated with increasing doses of brassinin (0, 20, 50 and 100 µM). To measure the protein concentration, a Bradford protein assay (Bio-Rad, Hercules, CA, USA) was performed. The denatured proteins were separated by sodium dodecyl sulfate-polyacrylamide gel electrophoresis (SDS-PAGE) and transferred to nitrocellulose membranes. Light intensity of the whole blots was measured using a ChemiDoc EQ system and Quantity One software (Bio-Rad) as previously described [16].

### 2.10. Quantitative Real-Time PCR

Total RNA of Huh7 and Hep3B was extracted by TRIzol reagent (Invitrogen, Carlsbad, CA, USA) after brassinin (0 and 100 µM) treatment for 24 h. AccuPower PreMix (Bioneer, Daejeon, Korea) and StepOnePlus Real-Time PCR system (Applied Biosystems, Foster City, CA, USA) were used for synthesizing complementary DNA. Experimental protocols were performed according to the manufacturer’s instructions. Primer information has been described in Table 2.

### 2.11. Statistical Analysis

All data results were subjected to analysis of variance following the general linear model (PROC-GLM) of the SAS program (SAS Institute, Cary, NC, USA). Differences with a probability value of * *p* < 0.05 were considered statistically significant. Data are presented as the mean ± SEM unless otherwise stated.

## 3. Results

### 3.1. Brassinin Regulates Proliferation and Cell Cycle in HCC Cells

**Figure 1:**
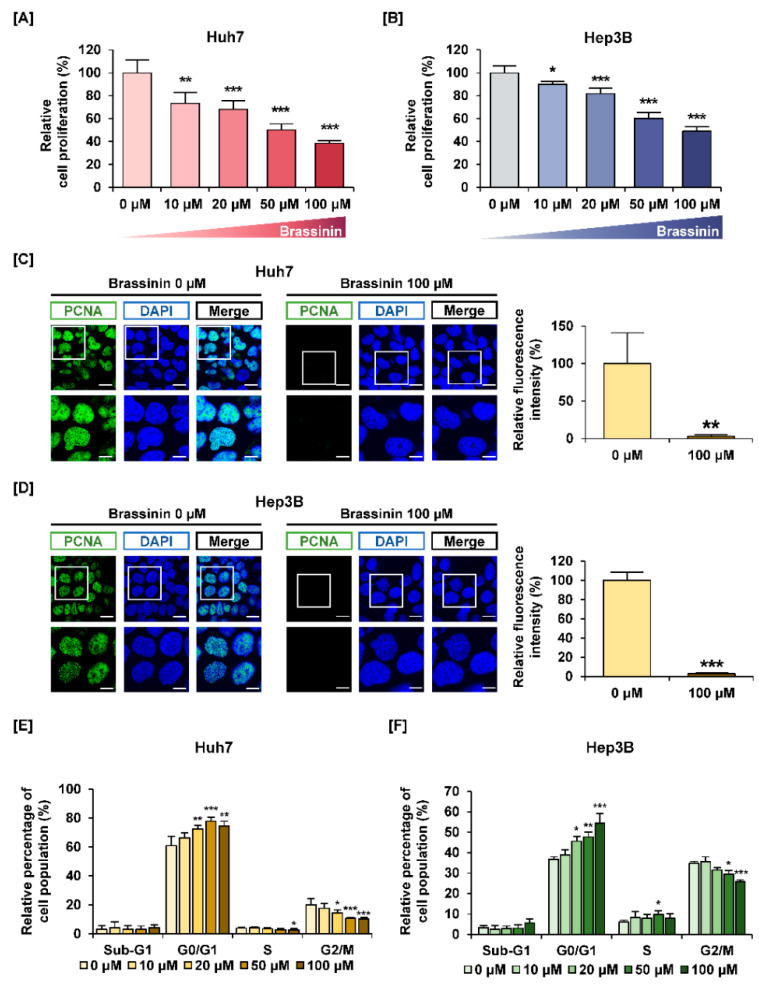
Effects of brassinin on proliferation and cell cycle of human hepatocellular carcinoma (HCC) cells. (**A**,**B**) The proliferation of Huh7 and Hep3B cells in response to brassinin. Results were compared to vehicle-treated cells. (**C**,**D**) Green fluorescence represents proliferating cell nuclear antigen (PCNA) and blue fluorescence represents DAPI as counterstaining for nuclei. Scale bar: 20 µm (top line) and 40 µm (bottom). (**E**,**F**) Cell cycle distributions. The graphs show the relative cell population compared to the control. Asterisks represent the significance levels between vehicle-treated cells and brassinin-treated cells (* *p* < 0.05, ** *p* < 0.01 and *** *p* < 0.001).

Brassinin reduced cell proliferation in a dose-dependent manner (Figure 1A,B). Specifically, 100 µM of brassinin reduced the proliferation of Huh7 cells to 39% and that of Heb3B cells to 49% (*** *p* < 0.001). In contrast, brassinin suppressed the viability of AML-12 cells (mouse normal liver cells) to about 86% compared with the vehicle, which implies that brassinin works specifically on HCC cells (Supplementary Figure S1A). We also compared the immunofluorescence intensity of PCNA between HCC cells treated with 100 µM brassinin and HCC cells that were untreated. Brassinin greatly reduced the relative intensity of PCNA in both Huh7 and Hep3B cells (Figure 1C,D). Then, we confirmed whether brassinin induces cell cycle arrest in Huh7 and Hep3B cells. Brassinin increased the relative proportion of cells in the G0/G1 phase in both cell lines (Figure 1E,F). It also significantly reduced the proportion of cells in the G2/M phase in both cell lines. In response to brassinin (0, 20, 50 and 100 µM), phosphorylation of CCND1 proteins gradually decreased in both Huh7 and Hep3B cells (Figure S1B). Also, *CDK2* mRNA expression was significantly suppressed by brassinin (100 µM), whereas *P21* mRNA expression was increased in both HCC cells (Figure S1C). These results indicate that brassinin suppresses the proliferation of Huh7 and Hep3B cells by arresting the cell cycle at the G0/G1 phase.

### 3.2. Brassinin Hampers Mitochondrial Homeostasis in Huh7 and Hep3B Cells

**Figure 2:**
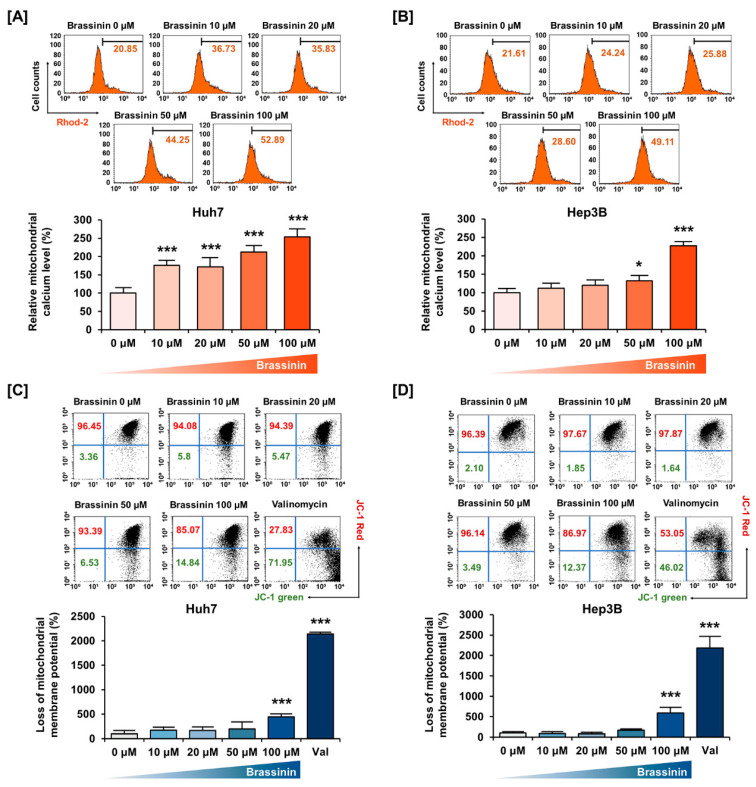
Changes in mitochondria calcium levels and mitochondrial membrane potential (MMP) caused by brassinin. (**A**,**B**) Mitochondrial calcium levels. Relative values indicated in the histogram are represented as a bar graph under the histogram. (**C**,**D**) MMP disruption. Val abbreviation stands for Valinomycin, the positive control. Asterisks represent the significance levels between vehicle-treated cells and brassinin-treated cells (* *p* < 0.05 and *** *p* < 0.001).

We assessed the relative levels of Ca^2+^ in mitochondria using Rhod-2 dye and the MMP using JC-1 dye (Figure 2). A dose of 100 µM brassinin increased the mitochondrial calcium ions concentration to 253% (*** *p* < 0.001) in Huh7 cells and 227% (*** *p* < 0.001) in Hep3B cells (Figure 2A,B). Also, brassinin increased the loss of MMP by 4.4-fold (*** *p* < 0.001) in Huh7 cells and 5.8-fold (*** *p* < 0.001) in Hep3B cells compared to the vehicle group (Figure 2C,D). Valinomycin (Val), the potassium ionophore, was used as a positive control for MMP. In addition, we performed western blot analysis for MMP-related proteins. In response to brassinin treatment (0, 20, 50 and 100 µM), phosphorylation of BAD and BCL-2 was decreased in Huh7 cells (Figure S3). Also, expression of BAK and BAX was increased in brassinin-treated Huh7 cells but the expression of MMP-related proteins in brassinin-treated Hep3B cells showed no significant changes (Figure S3). Taken together, these results indicate that brassinin disrupts mitochondrial homeostasis in Huh7 and Hep3B cells.

### 3.3. ROS Generation is Induced by Brassinin in Huh7 and Hep3B Cells

**Figure 3:**
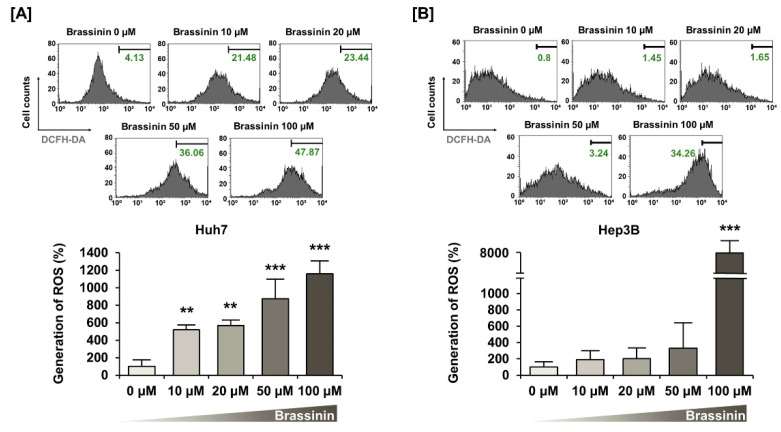
Effects of brassinin on reactive oxygen species (ROS) generation in HCC cells. (**A**,**B**) ROS production detected by brassinin-treated cells. Asterisks represent the significance levels between vehicle-treated cells and brassinin-treated cells (** *p* < 0.01 and *** *p* < 0.001).

**Figure 4:**
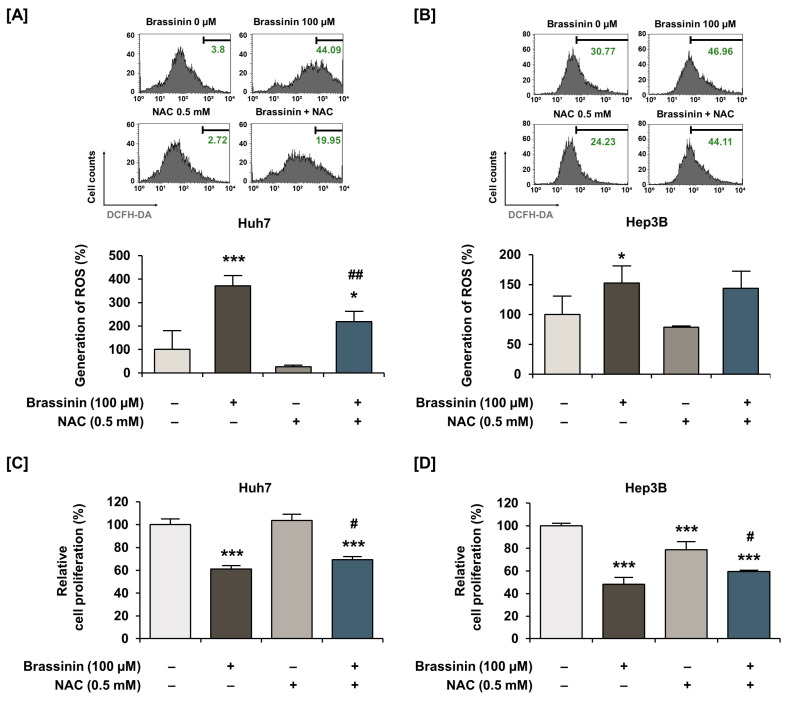
Effects of N-acetyl-L-cysteine (NAC) on brassinin-induced ROS generation and antiproliferative effects in HCC cells. (**A**,**B**) ROS generation by cells treated with N-acetyl-L-cysteine (NAC, a ROS scavenger, 0.5 mM), brassinin (100 µM) or both. (**C**,**D**) Proliferation of cells treated with N-acetyl-L-cysteine (NAC, a ROS scavenger, 0.5 mM), brassinin (100 µM) or both. Asterisks represent the significance levels between vehicle-treated cells and NAC-treated cells (* *p* < 0.05 and *** *p* < 0.001). In addition, # indicates significant differences as compared with brassinin alone (# *p* < 0.05 and ## *p* < 0.01).

Buffering dramatic changes in oxidative stress is one of the crucial functions of mitochondria. Thus, to measure the generation of ROS in HCC cells, we stained cells using DCFH-DA. Brassinin strongly increased ROS production by 11-fold (*** *p* < 0.001) in Huh7 and 74-fold (*** *p* < 0.001) in Hep3B (Figure 3A,B) cells. Compared with HCC cells, the brassinin treatment did not significantly change ROS generation in AML-12 cells, which implies that brassinin specifically induces excessive ROS generation in cancer cells (Figure S2). Then, we confirmed the ROS production in both HCC cell lines by brassinin with or without NAC, a ROS scavenger. NAC significantly reduced the brassinin-induced ROS production from 371% to 219% in Huh7 cells (Figure 4A) and slightly decreased it from 152% to 143% in Hep3B cells compared to vehicle-treated cells (Figure 4B). To demonstrate whether brassinin-induced ROS could affect the proliferation of HCC cells, we compared the proliferation of cells treated with brassinin, NAC or both (Figure 4C,D). NAC restored the brassinin suppressed proliferation of Huh7 and Hep3B cells. Collectively, our results reveal that brassinin specifically decreases cell proliferation in HCC cells by increasing ROS production.

### 3.4. Brassinin Regulates PI3K and MAPK Signaling Pathway in HCC Cell Lines

**Figure 5:**
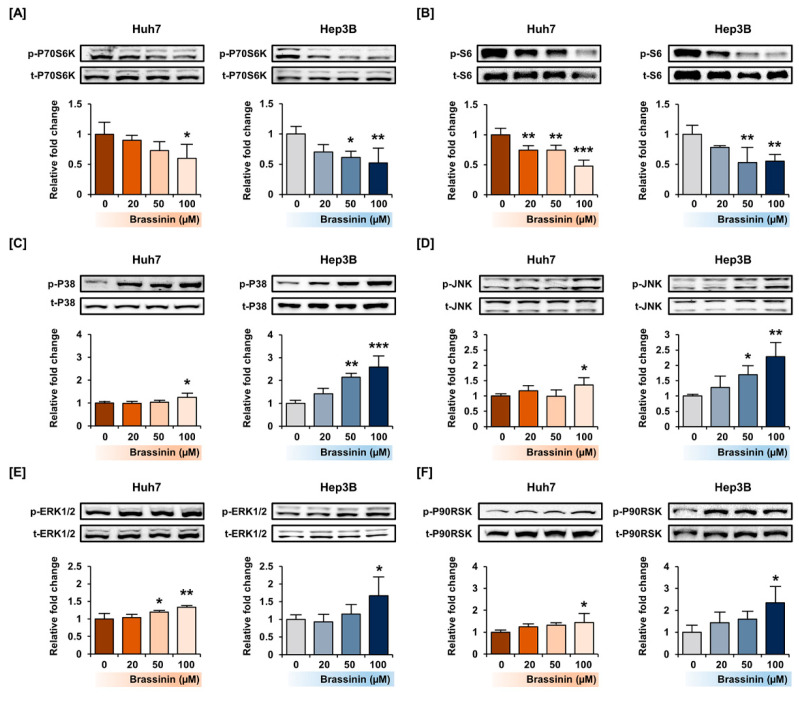
Activation of the PI3K/AKT and MAPK signaling pathways by brassinin in Huh7 and Hep3B cells. (**A**–**E**) Phosphorylation of P70S6K (**A**), S6 (**B**), P38 (**C**), JNK (**D**), ERK1/2 (**E**) and P90RSK (**F**). The standardized values of the phosphorylated proteins were estimated relative to the amount of total protein. Asterisks represent the significance levels between vehicle-treated cells and brassinin-treated cells (* *p* < 0.05, ** *p* < 0.01 and *** *p* < 0.001).

To confirm the phosphorylation of signaling proteins by brassinin, we conducted western blot analysis focused on the PI3K and MAPK pathways. Brassinin dose-dependently downregulated the phosphorylation of the P70S6K and S6 proteins, which are downstream of the PI3K signaling pathway (Figure 5A,B). On the other hand, the phosphorylation of P38 and JNK increased in Huh7 cells (Figure 5C,D). Besides, brassinin slightly increased the phosphorylation of ERK1/2 and its downstream P90RSK in both Huh7 and Hep3B cells (Figure 5E,F). Overall, brassinin suppressed proteins downstream of the AKT signaling pathway and increased P38/JNK/ERK signaling in HCC cell lines.

### 3.5. Effects of MAPKs Pharmacological Inhibitors on the Proliferation of HCC Cells with or without Brassinin

**Figure 6:**
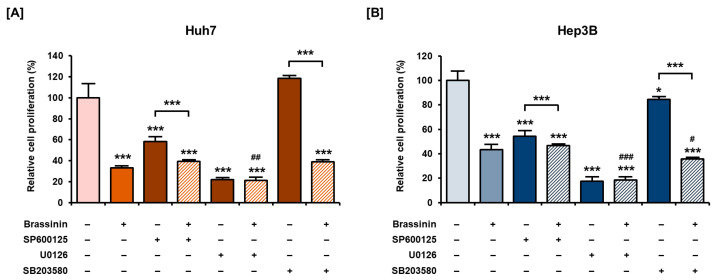
Antiproliferative effects of MAPK pharmacological inhibitors with or without brassinin in HCC cells. (**A**,**B**) Proliferation of cells treated with 20 µM SP600125 (JNK inhibitor), U0126 (ERK1/2 inhibitor) or SB203580 (P38 inhibitor) with or without brassinin (100 µM). Results were compared with vehicle-treated cells, brassinin-treated cells and each inhibitor-treated cells. Asterisks represent the significance levels between the vehicle-treated cells and the inhibitor-treated cells (* *p* < 0.05 and *** *p* < 0.001). # indicate the significance levels between brassinin-treated cells and cells treated with brassinin and each inhibitor (# *p* < 0.05, ## *p* < 0.01 and ### *p* < 0.001).

Next, we investigated the antiproliferative effects of MAPKs pharmacological inhibitors on HCC cells. SP600125 (JNK inhibitor) and U0126 (ERK1/2 inhibitor) alone, after treatment for 48 h, significantly suppressed the proliferation of Huh7 and Hep3B cells (Figure 6A,B). In Hep3B cells, the combination of brassinin with SB203580 also inhibited the proliferation more than brassinin alone (Figure 6B). Although there are no combined effects or restoration effects of brassinin with pharmacological inhibitors, we found that the regulation of MAPK is crucial for the proliferation of HCC cells (Figure 6). Overall, MAPK pharmacological inhibitors and brassinin had effective antiproliferative effects on HCC cells and revealed enhanced effects in certain combinations.

## 4. Discussion

In the present study, brassinin decreased cell proliferation, PCNA expression and MMP in human HCC cells. Also, brassinin increased mitochondrial calcium levels and ROS production in both Huh7 and Hep3B cells. The ROS scavenger NAC suppressed brassinin-induced ROS production and restored cellular proliferation in Huh7 and Hep3B cells. Thus, brassinin induced mitochondrial dysfunction and antiproliferative effects on HCC cells by regulating the MAPK and PI3K signaling pathways. Therefore, in this study, we elucidated the anticancer effects of brassinin on HCC cells and their cellular mechanisms.

ROS play important roles in energy metabolism, regulation of apoptosis and cell signaling during carcinogenesis [17]. ROS generation is inevitable because oxygen is converted to ROS through redox reactions during respiration to maintain proper ROS levels in cells. A loss of control of ROS generation in normal cells causes DNA damage due to free radicals and even leads to cancer [18]. Similarly, ROS are one of the causes of the development of nonalcoholic steatohepatitis (NASH) to HCC [19,20]. Usually, cancer cells produce more ROS due to their high metabolic and proliferation rates compared with normal cells but excessive ROS production can also kill cancer cells [21]. For instance, walsuronoid B induces cell apoptosis via ROS/p53-mediated mitochondrial depolarization and inhibits cell proliferation via G2/M phase arrest [22]. Homobrassinin, a brassinin derivative, has a ROS-dependent antiproliferative effect on human colorectal cancer cells (Caco2) and induces apoptosis through ROS generation and mitochondrial dysfunction [23]. In our study, brassinin induced excessive ROS generation in both Huh7 and Hep3B cells but NAC significantly suppressed ROS production in Huh7 cells, not in Hep3B cells. This might be explained by the difference of P53 phenotype and response to oxidant stimulus in Huh7 (mutated p53) and Hep3B (deleted P53) cells [24,25]. However, further studies are required to demonstrate a different sensitivity to NAC in Hep3B cells.

Mitochondrial calcium overload and depolarization of MMP are crucial checkpoints of cell death. Mitochondrial calcium levels are controlled by the mitochondrial calcium uniporter complex and the mitochondrial permeability transition pore [26,27]. In liver cancer, well-coordinated mitochondrial homeostasis promotes tumor growth [28]. However, an imbalance of calcium regulation in mitochondria causes mitochondrial swelling and rupture of the mitochondrial outer membrane [29]. Then, cytochrome c is released from mitochondria and initiates intrinsic-mitochondrial apoptosis. Maintenance of MMP is an indicator of mitochondrial bioenergetics as it is crucial for ATP synthesis and is deeply involved in ROS generation [29,30]. Therefore, a wide range of cancer treatments targets mitochondrial integrity. For instance, mitotane affects mitochondrial bioenergetics and induces apoptosis via mitochondrial membrane depolarization in thyroid cancer [31]. Also, upregulation of ROS and loss of MMP induce apoptosis in Hep3B cells by increasing phospho-JNK levels, which is consistent with our results [32]. Although MMP-related protein expression in Hep3B cells in response to brassinin did not show significant changes within 24 h, we speculate that it might further progress to the disruption of mitochondrial homeostasis, considering the results of Huh7 cells.

MAPKs of the serine/threonine kinases family play a role in apoptotic signaling and therefore in proliferation, gene expression, cell survival and apoptosis [33]. Thymoquinone induces the phosphorylation of the MAPK P38, which produces ROS and contributes to the antiproliferative and proapoptotic effects of this compound on breast cancer cells [34]. Also, a natural compound from Crataegus pinnatifida activates phospho-P38, which promotes autophagy and apoptosis in Hep3B cells [35]. Our results revealed that the antiproliferative activity of SB203580 was not evident in Huh7 cells, even though protein expression profiles were quite similar between Huh7 and Hep3B cells [36]. This may be attributed to the difference between the p53 phenotype between the two cell lines, considering the relationship between P38 and p53 in cancers [37]. Besides, increasing ROS generation in liver cells promotes the phosphorylation of JNK, which further stimulates ROS generation, forming a positive feedback loop [38]. Furthermore, blocking MAPKs using pharmacological inhibitors such as U0126 dramatically increased the antiproliferative effect of brassinin on HCC, which implies that MAPK signals are crucial for the survival of HCC cells. The PI3K signaling pathway regulates essential cellular functions such as proliferation, translation, survival and growth, as well as metastasis or invasion of cancer cells [39]. In HCC cells, the PI3K/AKT/FOXO4 signaling pathways are involved in cellular proliferation, tumor survival and other oncogenic processes [40]. Also, activation of P70S6K has a role in angiogenesis, which makes HCC cells more malignant and difficult to cure [41,42]. In our work, brassinin induced HCC cell cycle arrest at the G0/G1 phase. Likewise, brassinin inhibited PI3K signaling through the regulation of CDK inhibitors, leading to G1 phase arrest in colon cancer [43].

## 5. Conclusions

Overall, our study is the first to demonstrate the anticancer effects of brassinin on HCC cells and to elucidate their cellular mechanisms and action on mitochondrial function by activating MAPK pathways via ROS generation and MMP depolarization. Also, we confirmed that brassinin suppressed proliferation via ROS generation in liver cancer cells, without affecting the normal cells. This might suggest that brassinin could selectively eliminate cancer cells in the liver, without affecting healthy cells. Although our results are limited to an in vitro evaluation, this study may provide valuable insights regarding the underlying molecular mechanisms for future in vivo studies.

## Figures and Tables

**Table 1:** Antibodies used in immunoblotting.

Antibodies	Catalog No.	Sources
*p*-P70S6K (Thr^421^/Ser^424^)	9204	Cell signaling Technology (Danvers, MA, USA)
*p*-S6 (Ser^235/236^)	2211	Cell signaling Technology
*p*-P38 (Thr^180^/Tyr^182^)	4511	Cell signaling Technology
*p*-JNK (Thr^183^/Tyr^185^)	4668	Cell signaling Technology
*p*-ERK1/2 (Thr^202^/Tyr^204^)	9101	Cell signaling Technology
*p*-P90RSK (Thr^573^)	9346	Cell signaling Technology
t-P70S6K	9202	Cell signaling Technology
*p*-CCND1 (Thr^286^)	3300	Cell signaling Technology
t-S6	2217	Cell signaling Technology
t-P38	9212	Cell signaling Technology
t-JNK	9252	Cell signaling Technology
t-ERK1/2	4695	Cell signaling Technology
RSK1/RSK2/RSK3	9355	Cell signaling Technology
*p*-BAD (Ser^112^)	5284	Cell signaling Technology
*p*-BCL-2 (Ser^70^)	2827	Cell signaling Technology
BAK	12105	Cell signaling Technology
BAX	2772	Cell signaling Technology
TUBA	Sc-32293	Santa Cruz Biotechnology

**Table 2:** Primers used in quantitative reverse transcription polymerase chain reaction (qRT-PCR).

Genes	Accession Number	Primer	Sequence (5′-3′)
*CDK2*	BT006821.1	Forward	AAATTCATGGATGCCTCTGC
		Reverse	GCCCCCTCTGTGTTAATAAGC
*P21*	NM_000389.5	Forward	GACTCTCAGGGTCGAAAACG
		Reverse	GGATTAGGGCTTCCTCTTGG
*GAPDH*	NM_001256799.3	Forward	ACCCAGAAGACTGTGGATGG
		Reverse	TGACAAAGTGGTCGTTGAGG

## Data Availability

Data available in a publicly accessible repository.

## Supplementary Materials

The following are available online at https://www.mdpi.com/2073-4409/10/2/332/s1, Figure S1: Effects of brassinin on normal liver cells (AML-12) and cell cycle progression (HCC cells); Figure S2: Effects of brassinin on ROS generation in AML-12 cells. (A) ROS production detected by brassinin-treated AML-12 cells; Figure S3: Protein expression changes about mitochondria-membrane related proteins in brassinin-treated Huh7 and Hep3B cells.
