# Supplementary material for: Brassinin Inhibits Proliferation in Human Liver Cancer Cells via Mitochondrial Dysfunction
Source: Cells. 2021 Feb 5;10(2):332. doi: 10.3390/cells10020332 (PMC7915448; doi:10.3390/cells10020332)
Supplement: Supplementary file 1 [file cells-10-00332-s001.pdf]

cells

Supplementary Materials

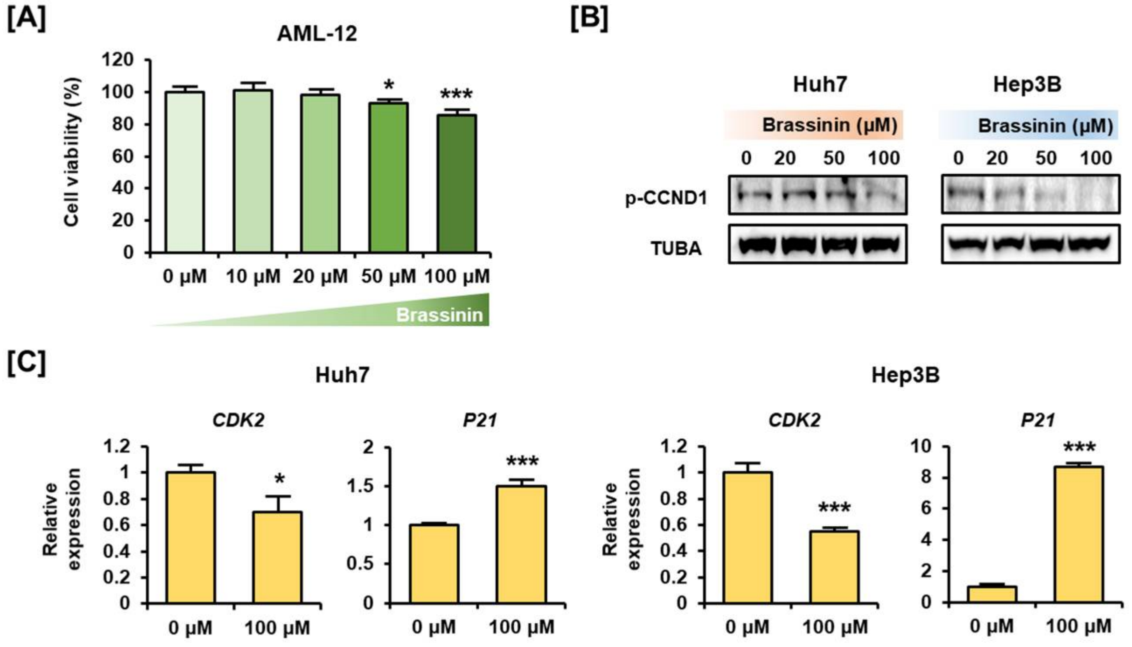

Figure S1. Effects of brassinin on normal liver cells (AML-12) and cell cycle progression (in HCC cells). (A) Viability of AML-12 cells, normal mouse liver cells, in response to brassinin. Viability was evaluated using the MTT assay. (B) Immunoblots of phosphorylation of Cyclin D1 (CCND1) proteins in response to brassinin (0, 20, 50, and 100 μM) on Huh7 and Hep3B cells. α-tubulin (TUBA) was used for normalization and protein quantification. (C) mRNA expression levels of *CDK2* and *P21* in response to brassinin (100 μM) on HCC cellsanalyzed by quantitative RT-PCR. The asterisks represent significance levels between vehicle-treated and brassinin-treated cells (*p < 0.05 and ***p < 0.001).

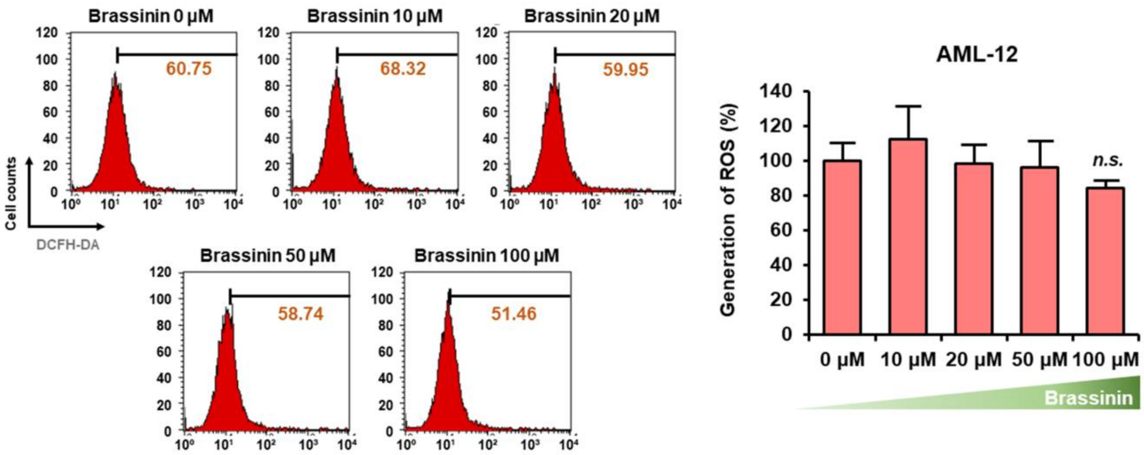

Figure S2. Effects of brassinin on ROS generation in AML-12 cells. ROS production in brassinin-treated AML-12 cells. n.s. stands for non-significant difference between vehicle- and brassinin-treated cells.

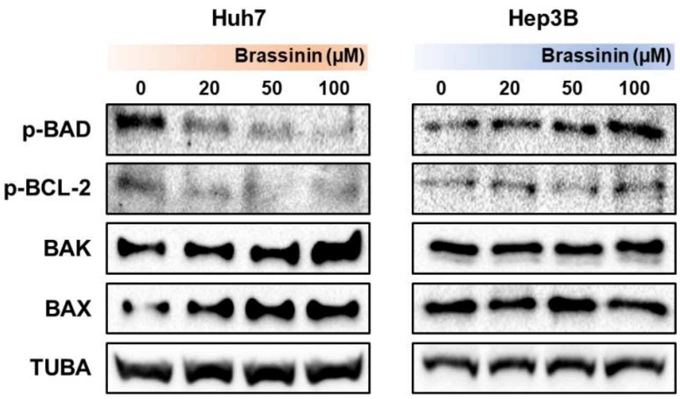

Figure S3. Protein expression changes in mitochondrial membrane-related proteins in brassinin-treated Huh7 and Hep3B cells. Phosphorylation of BAD and BCL-2 and quantities of BAK and BAX in response to brassinin treatment were elucidated from immunoblots. Brassinin (0, 20, 50, and 100 μM) treatments were performed for 24 h in Huh7 and Hep3B cells. TUBA was confirmed as a loading control for mitochondrial proteins.
